# Knowledge brokers in a knowledge network: the case of Seniors Health Research Transfer Network knowledge brokers

**DOI:** 10.1186/1748-5908-8-7

**Published:** 2013-01-09

**Authors:** James Conklin, Elizabeth Lusk, Megan Harris, Paul Stolee

**Affiliations:** 1 Care of the Elderly Research Program, Bruyère Research Institute, 43 Bruyère Street, K1N 5C8, Ottawa, ON, Canada; 2 Department of Applied Human Sciences, Concordia University, 1455 de Maisonneuve Blvd. West, H3G 1M8, Montréal, QC, Canada; 3 Gestalt Collective, 79 Dewar Court, L9T 5N8, Milton, ON, Canada; 4 School of Kinesiology, Faculty of Health Sciences, Western University, N6A 5B9, London, ON, Canada; 5 Canadian Dementia Resource and Knowledge Exchange, Windsor, Canada; 6 Alzheimer’s Knowledge Exchange, 20 Eglinton Avenue West, Suite 1600, M4R 1K8, Toronto, ON, Canada; 7 School of Public Health and Health Systems, Faculty of Applied Health Sciences, University of Waterloo, 200 University Ave, West, N2L 3G1, Waterloo, ON, Canada; 8 Schlegel-University of Waterloo Research Institute for Aging, 325 Max Becker Drive, Suite 202, N2E 4H5, Kitchener, ON, Canada

**Keywords:** Knowledge broker, Knowledge exchange, Knowledge translation, Community of practice, Knowledge network

## Abstract

**Background:**

The purpose of this paper is to describe and reflect on the role of knowledge brokers (KBs) in the Seniors Health Research Transfer Network (SHRTN). The paper reviews the relevant literature on knowledge brokering, and then describes the evolving role of knowledge brokering in this knowledge network.

**Methods:**

The description of knowledge brokering provided here is based on a developmental evaluation program and on the experiences of the authors. Data were gathered through qualitative and quantitative methods, analyzed by the evaluators, and interpreted by network members who participated in sensemaking forums. The results were fed back to the network each year in the form of formal written reports that were widely distributed to network members, as well as through presentations to the network’s members.

**Results:**

The SHRTN evaluation and our experiences as evaluators and KBs suggest that a SHRTN KB facilitates processes of learning whereby people are connected with tacit or explicit knowledge sources that will help them to resolve work-related challenges. To make this happen, KBs engage in a set of relational, technical, and analytical activities that help communities of practice (CoPs) to develop and operate, facilitate exchanges among people with similar concerns and interests, and help groups and individuals to create, explore, and apply knowledge in their practice. We also suggest that the role is difficult to define, emergent, abstract, episodic, and not fully understood.

**Conclusions:**

The KB role within this knowledge network has developed and matured over time. The KB adapts to the social and technical affordances of each situation, and fashions a unique and relevant process to create relationships and promote learning and change. The ability to work with teams and to develop relevant models and feasible approaches are critical KB skills. The KB is a leader who wields influence rather than power, and who is prepared to adopt whatever roles and approaches are needed to bring about a valuable result.

## Background

The purpose of this paper is to contribute to our understanding of knowledge brokering in Canada’s health system. We offer a description of knowledge brokering in the Seniors Health Research Transfer Network (SHRTN), drawing on the results of a five year evaluation program carried out by the network, and also on the experiences of two members of our authoring team (EL and MH) as KBs in the network. The article comments on the roles, skills, and potential impact of knowledge brokers (KBs) in the network.

SHRTN was formed in 2005 to encourage the sharing of scientific and practice-based knowledge in Ontario’s seniors health system [1]. Supported by the Ontario Ministry of Health and Long Term Care, led by a Board of Directors consisting of researchers, practitioners, and policy makers, and managed through a Secretariat, SHRTN has become a thriving knowledge network recognized for its contribution to the health and wellbeing of Ontario’s seniors [2,3].

Now known as the Seniors Health Knowledge Network, the network functions as a ‘network of networks’ consisting of three partners. The SHRTN Knowledge Exchange links caregivers, researchers, and policy makers who seek to improve knowledge exchange within Ontario’s seniors health system. The Alzheimer Knowledge Exchange (AKE) promotes knowledge sharing among researchers, policy makers, educators, care providers, and stakeholders working on dementia-related issues. The Ontario Research Coalition of Research Institutes on Health and Aging brings together institutes that conduct research to improve the quality of care of Ontario seniors. This article relies on data gathered about KBs in the SHRTN Knowledge Exchange. For the balance of this article, we refer to these KBs as ‘SHRTN KBs.’

When SHRTN was launched, little was known about knowledge networks. The Institute for Sustainable Development had published its report on the use of knowledge networks to promote sustainable development [4]. Provan and Milward had published their article on the levels of social impact that can be realized through a network [5]. The Canadian Health Services Research Foundation (CHSRF) had begun to investigate the factors that underpin the success of knowledge networks [6]. However, there was no blueprint for successful knowledge networks.

As a result, SHRTN leaders took a developmental approach to building their network. In its first year, SHRTN established governance and leadership processes, and a series of projects that allowed stakeholders to become involved. At the end of this inaugural year, word about the network was spreading through the participation of organizations such as the Ontario Long-Term Care Association.

In its second year, the network supported a small number of network components that would promote the growth and sustainability of the network. These components included a library service to make knowledge more accessible, communities of practice (CoPs) to bring together people working on similar issues, and KBs to strengthen relationships throughout the network.

In this paper we explore two questions about the KBs: What roles and skills characterize SHRTN KBs? And to what extent is the KB role contextual and adaptive?

We present the case of knowledge brokering in SHRTN in the following ways. First, we summarize some views of knowledge brokering evident in the literature on the subject. We then offer background on the SHRTN evaluation program, which we used as a basis for this descriptive report. We then present lessons learned through the evaluation program, and we conclude with our thoughts on the importance of knowledge brokering for bringing innovations into frontline healthcare organizations.

### The theory and practice of knowledge brokering

The need for a role within the health system to facilitate knowledge transfer and exchange (KTE) stems from the conviction that many barriers impede the effort to introduce change into healthcare contexts [7]. Some believe that the key to overcoming these barriers lies in techniques for changing human behaviour [8]. This has led to a focus on roles that facilitate interactions across the social boundaries that characterize our health system. Management scholars [9-13] and health services researchers [6,14-16] have considered whether knowledge brokering is a role that can facilitate the transfer of knowledge across social boundaries.

Our review of the management and health services literature suggests that KBs are often associated with seven types of activity. These include activities intended to:

1. Create relationships among groups of people with shared concerns and objectives;

2. Promote mutual understanding among these groups;

3. Facilitate the exchange of knowledge across the social boundaries that separate these groups;

4. Facilitate processes of social interaction as a mechanism for bringing about knowledge exchange;

5. Develop new capacity within these groups to work together to find, create, share, and use relevant knowledge;

6. Help to address the issues of organizational change that often accompany attempts to exchange knowledge;

7. Engage in analytical tasks that are associated with the above activities.

KBs have been referred to as ‘boundary spanners’ [17] or ‘bridge builders’ [14,18,19]. They link researchers who produce scientific knowledge and practitioners who produce experience-based knowledge with knowledge users (including decision makers and caregivers) [20-22]. KBs are also seen as fostering relationships and creating operational groups capable of producing tangible results, and as ensuring the ongoing health and success of network alliances [23,24]. Some suggest that connecting people is the core function of KBs, and that this requires establishing trusting relationships that encourage conversations about the introduction of changes into frontline practices [25,26]. An effective KB implements a strategy for forming partnerships and engaging stakeholders [27].

The KB role is also seen as promoting mutual understanding that gives researchers, decision makers, and caregivers a better understanding of each others’ environments and cultures, and that helps to spread the awareness and adoption of innovations [14,22,28,29]. This focus on promoting a shared understanding is also seen as fostering commitment among key stakeholders [30]. KBs link decision makers with researchers so they can understand each others’ goals, cultures, and constraints, and can thus collaborate on how best to use evidence in decision making [31]. Not surprisingly, then, some point to the importance of developing trust and common ground [25,26], empathy [23], and interpersonal communication [27] for successful knowledge brokering.

KBs also engage in activities related to finding, appraising, transforming, and disseminating relevant knowledge that allows for improved performance [21,22,31-34]. This involves facilitating the creation of new knowledge to fill gaps [21,22]. It can also involve identifying knowledge sources, assessing the usefulness of knowledge, adapting it for local contexts, synthesizing knowledge into usable formats, and finding meaningful roles for knowledge users to play in the research process [15,23,29,35-38].

The literature also makes it clear that KBs are active within the social groupings where they operate. Their work involves facilitating social interaction [39] and collaborative processes [37,38] that often focus on activities intended to find, assess, interpret, and adapt the evidence people are seeking, and to identify emerging issues that could be resolved by the use of research knowledge [36].

These KB activities aim at solving immediate problems and creating new capacity [21,22]. This is evident in the emphasis on facilitating skill development and adult education that is prominent in some studies [15,35], and on teaching and mentoring activities [22,29]. KBs may also be involved in organizational change [15,35]. One reason for bringing people together through the work of KBs is to foster conversations about the introduction of change and improvement into frontline practices [25,26].

KBs often undertake a variety of analytical and reflective activities. For example, KBs periodically conduct needs assessments to identify the knowledge resources needed to solve compelling frontline challenges [23]. Others have pointed out that successful KBs engage in critical thinking and reflective practice [15].

Given the diversity of KB practice, many suggest that knowledge brokering is both complex and contextual. Some argue that although interactive engagement is a common feature of knowledge brokering, the KB role is contextual and thus it is difficult to posit a standardized set of KB skills [40,41]. Others acknowledge that although KBs use similar skills, each KB uses these skills in different ways in different situations [22,29]. Some have argued that knowledge brokering is surprisingly complex, and that KBs must continuously assess needs and adapt their activities to emerging situations [15,35].

### Knowledge brokering in SHRTN: the espoused role

Some argue that the effectiveness of a knowledge network can be enhanced through the work of KBs [1,2,42]. As indicated earlier, an important element in the design of SHRTN is the use of KBs to facilitate knowledge exchange across practice boundaries. Over the past several years, SHRTN has accumulated significant experiential knowledge concerning the role. In addition, SHRTN’s evaluation program has gathered information about the role of KBs in the network. By bringing this experience and knowledge to light, we hope to contribute to the understanding of the KB role.

Drawing on the 2003 CHSRF report, and through consultation with stakeholder groups, in 2006 SHRTN developed a formal job description for KBs that outlined several domains of activity. The KBs were expected to network and communicate, to promote and publicize the network, and to manage the network’s web-based tools. They were expected to identify useful knowledge and to facilitate the movement of that knowledge into practice. They were expected to work in partnership with leaders of SHRTN CoPs. This meant that they could be called upon to help CoPs form working groups, to educate these groups on knowledge translation concepts, to help the CoPs to develop goals and workplans, and to help CoP teams to implement their plans. They were also expected to foster partnerships throughout the network, coordinate ongoing communications, help CoPs to put on educational events, and support CoP members.

In addition to these CoP-level responsibilities, the KBs were also to play a role in the broader network. This included bringing the ideas and activities of the CoPs into the network in ways that reduced duplication and encouraged collaboration. They were expected to link stakeholders with the best available evidence and tools on knowledge exchange. Finally, they were to help coordinate, plan, and develop network processes, policies, and reporting structures.

We believe that we may be able to shed light on the role of knowledge brokering for two reasons. First, one way in which SHRTN explored what it means to be a knowledge network was through a mixed-methods developmental evaluation program [43,44]. Two of the authors (JC, PS) led this evaluation program over a period of five years, generating insights and conclusions about the role and significance of knowledge brokering [1,3]. The other authors (EL, MH) acted as KBs within SHRTN and similar networks for five years, and their experiences informed their contribution to this article.

## Methods

The SHRTN evaluation program was designed as a developmental evaluation program [43,44] intended to generate information that could be used by network leaders to achieve SHRTN’s objectives. Data were gathered through a variety of methods, analyzed by evaluators, and also interpreted by network members who participated in sensemaking forums. Results were fed back to the network in the form of written reports, as well as through presentations to the network’s governing body and to members at provincial and regional meetings.

The developmental intent of the evaluation program meant that the evaluation focused on the growth and improvement of the network, and not on generating new scientific knowledge or on a definitive assessment of impact. This paper therefore describes and comments on the KB role in a specific social milieu, and should not be seen as a template for knowledge brokering that could be implemented in all social environments. Our purpose is to contribute to the growing understanding of knowledge brokering—what it is, what it consists of, in one specific social milieu—by describing the KB role as it unfolded within SHRTN.

During its first year, SHRTN formed an evaluation advisory group and hired two evaluators to design and implement the program. In this first year, SHRTN’s contract with the Ministry was based on a set of deliverables that network leaders were expected to achieve, and hence much of the evaluation work focused on these deliverables (there were no KBs working in the network in this first year).

In its second year, SHRTN based its work on three pillars: KBs, CoPs, and a library service. During this year, the evaluators assessed the work of the new KBs, CoPs, and library service; they devised a method for testing SHRTN’s effectiveness as a knowledge-exchange system, and ran one test using the method; and they created an overall evaluation approach for SHRTN. In developing an evaluation framework, the Promoting Action on Research Implementation in Health Services (PARiHS) model was adopted as a theoretical lens to guide the evaluation [3,45]. PARiHS gave the evaluators and SHRTN leaders a shared perspective on three key dimensions of knowledge translation: the evidence that the network is attempting to move across social boundaries; the facilitation techniques used to encourage the movement of this evidence; and the social context that is expected to adopt and use the evidence. Data was gathered and analyzed in relation to these three dimensions, and when SHRTN leaders and evaluators met to discuss the findings, these discussions were often organized in terms of the PARiHS dimensions. The evaluation approach included a case study method that focused on specific instances of knowledge moving through the network.

The SHRTN year three evaluation program involved surveys, reviews of SHRTN reports and other documents, and two detailed case studies examining specific knowledge exchange initiatives. In accordance with the agreed-to evaluation approach, the objective was to assess the development of the network in relation to linkages and knowledge exchange, the awareness and perceptions of value of SHRTN, the movement of new knowledge into practice, and progress against the explicit targets set by SHRTN leaders.

The evaluation that began in year four continued into the network’s fifth year, covering approximately 18 months of activity. The objectives focused on the four broad areas of linkage and exchange, awareness and perceptions of SHRTN’s value, specific cases of the movement of knowledge into practice, and progress against explicit goals. The SHRTN board asked the evaluators to gather information about the CoPs that were operating in the network, investigate the role played by KBs, conduct case studies of knowledge translation, administer a survey of the community care sector, and inquire into various ways in which network participants conceived of ‘membership’ in the network. The evaluators were also to inquire into the value of the library service, and to gather data from people who worked at the network’s Secretariat.

To prepare this paper, we used the data concerning knowledge brokering and KBs that were gathered during year two, year three, and years four and five. Details concerning the overall data collection can be found in Additional file 1, and a list of the data sources pertaining to knowledge brokering can be found in Additional file 2.

A variety of techniques were used to organize and analyze the data. Documents were reviewed by one analyst, with summary notes used to extract key points. We reviewed documents such as the SHRTN strategic plan, meeting minutes, contracts and job descriptions for KBs and librarians (which allowed us to identify annual performance targets for these job categories). From these documents we created a grid of the goals, intentions, and objectives of SHRTN leaders, and we then compared this grid to information about accomplishments and challenges found in progress reports submitted by CoPs, KBs, and librarians. Narrative data from interviews and focus groups were analyzed by at least two and as many as five analysts, with findings compared and synthesized. Analytical methods included the constant comparison method and narrative coding and theming techniques [46,47]. Care was taken to ensure that all themes were well-anchored in the data (this was done by identifying the number of instances of individual codes, and the number of data sources supporting the codes). The results of analytic procedures were compared, to ensure that synthesized answers and themes were consistent, and that all themes had been identified.

For the KB investigation in years four and five, one evaluator read through the focus group and interview data, making notes about impressions and findings. Findings were then consolidated in relation to each question asked in the focus group and interviews. The evaluator wrote a narrative about the role, skills, and importance of the SHRTN knowledge brokers, drawing on the various data sources and interim analyses as needed. A second evaluator reviewed the narrative and data and suggested changes. The first evaluator considered these suggestions and then reviewed the supplementary documents that had been provided by informants, considering whether they were consistent with the findings, and making additional notes. The evaluator then wrote the narrative for the evaluation report, and itemized the points to include in a presentation to SHRTN stakeholders.

To analyze case study data, one evaluator read all data sources, making notes about the background to the case, and about the activities that occurred in the case. These notes became the basis for the narrative description of the case. As a member-checking step, the evaluator then sent the narrative to the CoP leaders and invited them to suggest corrections or additions. After finalizing the narrative description, the evaluator organized the interview data by arranging all answers in relation to questions, and removing identifiers. The evaluator then reviewed all qualitative data sources, and coded and themed the data using standard qualitative analytical techniques [46-50]. A second analyst reviewed these results, and suggestions were discussed.

To prepare this article, we reviewed evaluation findings for year two, year three, and years four and five, and extracted information related to the KBs. We organized these findings sequentially, to see if a developmental path was evident. We also organized the findings in relation to roles and skills evident in SHRTN KBs, and looked for findings that spoke to the contextual nature of their work and the value they provide.

## Results

From its second through to the end of its fifth year of operation, SHRTN stakeholders came to value the role and contribution of the network’s team of KBs, even if the nature of the role seemed elusive. During the network’s fifth year, one SHRTN leader remarked, ‘I think the KBs are essential to our task. They are at the core of knowledge translation, because at the end of the day it is those human relationships that bring about the most dynamic change. You need people with skills to enable groups to come together.’

During the network’s second year, the KB role went through a period of development and clarification. The evidence suggests that the KBs immediately made a useful contribution to at least five CoPs that were active in the network, and a more limited contribution to three other CoPs (the data was unclear on the contribution made to the remaining three CoPs). The KBs noted that they were most successful when supporting CoPs that were new and that were not based on pre-existing groups. During year two the KBs designed and facilitated workshops and knowledge exchange sessions, networked with CoPs and external organizations, conducted needs assessments, attended meetings and conferences to publicize the CoPs, participated in meetings with CoP members, assisted with CoP strategic planning, supported membership growth, developed tools, updated websites, and connected people who were working on similar problems.

The KBs indicated that the most effective way to engage stakeholders was through educational webinars, as well as through networking with decision makers, traditional workshops, focus groups, targeted recruitment of early adopters, and phone contacts. KBs also indicated that how they supported CoP activities depended on the CoP’s stage of development. When a CoP was in the early stages of development, the KB was involved in planning, facilitation, communication, sending invitations, conducting online searches, developing mailing lists, making phone calls to form relationships, and coaching CoP members in the use of online tools. For well-established CoPs, the KB performed fewer administrative tasks, and played more of a mentoring role with CoP leaders.

Most KBs described their primary role as connecting people with other people, information, ideas, and education. Most acknowledged that they also played a coordination role, and some indicated that they spent considerable time supporting the network’s web-based software. The job description for KBs in the network’s second year mentions networking and communication, promotion, management of online tools, identification of evidence, and facilitation. When asked about the importance of these roles, the KBs provided numerous examples of all of these responsibilities, with the exception of ‘identification of evidence.’ Though some KBs helped to identify evidence, most reported that this was a minor role. When asked which role occupied most of their time, KBs indicated that facilitation, networking and communication were their most significant roles.

Facilitation activities that KBs carried out included facilitating discussions and focus groups and chairing teleconferences. Examples of networking and communication activities included connecting with professional groups, attending meetings and conferences, writing articles, publishing newsletters, identifying and meeting with people who could benefit from the CoPs, communicating with members and stakeholders, supporting online knowledge exchange, and collaborating with other KBs.

When asked about her most important role, one KB said ‘Each of the CoPs I work with are in the early stages of development, therefore networking and communication contributed the most to the current successes being accomplished by these groups.’ A second KB suggested that the most important roles were ‘networking, supporting project work, in addition to knowledge brokering by phone and addressing email concerns in a timely manner.’ When looking toward the future of the KB role, one KB said that it was important to ‘continue to support strategies that increase awareness of the [network]; increase potential outcomes; and increase use [of the network].’ A second KB suggested that ‘the knowledge broker CoP needs time to work together and develop our own terms of reference and best practice guidelines related to working with CoPs.’ This latter point referred to the creation of a KB CoP as a way to share challenges and successes and to problem solve together. These statements suggest a focus on bringing people together to experience the value of participating in a CoP and a knowledge network, and on finding ways to support the growth of the CoPs.

During its third year, the network employed six KBs and supported thirteen CoPs. Network membership grew by 1,101, reaching 3,048 by the end of the year, and a total of 183 knowledge exchange events reached an audience of 4,023 participants. The year three evaluation focused more on knowledge exchange and the growth and performance of the CoPs, and less on the KBs. Nonetheless, KBs used a reporting template to share their perspectives, and commented on the continuing evolution of their role. KBs talked about disseminating information to CoP participants, facilitating discussions and meetings, handling logistical setup for meetings, preparing materials for presentations, troubleshooting technical problems, assisting with membership growth, and helping with the preparation of knowledge translation tools. One KB indicated that there was the potential for focusing the KB role on developing new practices and knowledge. Another said that some CoPs appeared to benefit from KB support more than others, and suggested that those CoPs that already had a strong sense of purpose and an action plan were less likely to need KB support.

When asked to share insights on effective ways of moving knowledge into practice, one KB responded, ‘since knowledge transfer in health care is relatively new, knowledge brokers can benefit from using models from the peer-reviewed literature to guide practice.’ The KBs also felt that it was important to be pragmatic and positive, and focus on using knowledge transfer to bring tangible improvements. One KB advised, ‘Keep things small, and celebrate small successes.’ A second suggested ‘placing knowledge transfer activities within the context of a performance improvement model that will help focus resources on closing gaps.’ A third said that knowledge brokering ‘is still a work in progress.’ The KBs emphasized the importance of allowing for interaction through enabling technology. One pointed out that ‘relative isolation and wide geographical dispersion of members makes use of existing SHRTN technology very attractive’ to caregivers, and a second said that the ‘ease of use of [SHRTN’s web-based technology] will greatly facilitate the achievement of objectives.’

In its fourth year, when the network employed five KBs and supported nineteen CoPs, network leaders explicitly sought to understand the KB role and the competencies needed to fill the role. The evaluation team was asked to define knowledge brokering—at least as it was practiced and experienced within this network. They were asked to gather information about the ‘doing’ of knowledge brokering, to catalogue recent KB accomplishments, and to identify the KB’s core skills.

### Defining knowledge brokering in SHRTN

SHRTN KBs facilitate processes of learning whereby people are connected with tacit or explicit knowledge sources that will help them to resolve work-related challenges. To make this happen, KBs help CoPs to develop and operate, facilitate exchanges among people with similar concerns and interests, and help groups and individuals to create, explore, and apply knowledge in their practice. The SHRTN experience also suggests that the role is evolving and is difficult to define.

One CoP leader said, ‘A KB is an individual who facilitates a process of dialogue between different parties who possess different knowledge, to find common ground between these parties to allow them to move forward with their collective work.’ Another said, ‘We are trying to promote a more integrated healthcare system. That is ultimately what we are doing. Building bridges, linking people, improving the flow of information to improve practice and to improve integration in the system.’

### The ‘doing’ of knowledge brokering

To provide network leaders with a perspective on the core skills of KBs, the evaluation team created four sets of narrative data: interview responses from CoP leaders who were asked to comment on KB activities and accomplishments; interview responses from CoP leaders who were asked to identify the core skills needed by successful KBs; interview responses from CoP leaders who were asked about the ways that KBs facilitate the flow of knowledge through the network; and focus group data from KBs who were asked about their accomplishments. Additional file 3 provides a summary of the key findings from each of these data sets.

When asked what KBs do and what they have accomplished within SHRTN, we received responses that highlight the contextual nature of the role. One CoP leader said ‘Our KB’s manner is conducive to facilitation and coaching. The KB … coaches people and fosters a lot of questions in a supportive way, and comes across as very open minded and supportive.’ Another mentioned a variety of activities and contributions: ‘Culture receptivity, improving readiness, finding the right people, fostering the process by which there is knowledge in the right formats. And cataloguing things that could be useful.’ Others talked about how the KBs ‘Work together to develop new and existing CoP and cross-CoP collaborations,’ and ‘bring information back from the CoPs on needs and barriers to help improve SHRTN.’

SHRTN KBs support their CoPs by providing coordination and administrative support, identifying knowledge resources, linking CoP members to those resources, creating relationships and dialogue, and fostering the growth of new CoPs. They also facilitate the flow of knowledge through the network by engaging in analytical and planning activities, coaching and supporting people, expanding existing networks, translating knowledge so it can be used by specific groups, planning and facilitating meetings and events, and handling administrative functions. SHRTN KBs must also possess excellent verbal and written communication skills, including the ability to influence and persuade others. They must be skilled at working with people and groups (including the ability to collaborate, direct, empower, support, and negotiate). A KB must have subject matter and technical skills (facilitation, project management, organizational change, research, learning, and project management). KBs must also possess personal qualities that equip them to work in ambiguous social environments (including time management, flexibility, and persistence).

KBs and those they support agree that KBs draw on their own experiences, and share their knowledge with each other and with others in SHRTN to promote the development and success of SHRTN CoPs. They also agree that KBs provide important information to SHRTN leaders, so solutions can be developed at a system-wide level, and so important information is available as leaders set a direction for the network.

## Discussion

SHRTN KBs promote knowledge exchange by locating and appraising knowledge, assessing the readiness of people to use new knowledge, and creating plans to help people to achieve their goals. Social learning theorists [51-53] suggest that people learn in workplace situations through a collective innovation process consisting of phases of action, reflection upon the action, analysis of opportunities revealed through the reflection, development of solutions and plans, and then implementation of plans through further action. This is similar to the conception of Wenger *et al.*[54] of a multi-membership learning cycle, where people participate in a learning loop that encompasses frontline workgroups and CoPs. KBs might be seen as creating and sustaining this reflective process, supporting the analytical processes of practice members, helping to develop plans for implementing new approaches in frontline practices, and then facilitating the movement of innovations into the frontline practices.

The KB role within SHRTN developed and matured over time. Some facets of the role, for example facilitation and networking, have been characteristic of the KBs since they were first introduced. The KBs often facilitate meetings and larger events at which people discuss common problems and possible solutions. They also bring people together in new relationships in order to promote the growth of the CoPs, and to widen the network of people who are arranging themselves around common issues. Although some facets of the KB role have remained stable, others have changed. Initially, KBs connected people with new resources and ideas; over time, this role expanded into a more purposive planning and analytical role that includes conducting environmental scans and needs assessments, assessing readiness to change, and developing strategies to bring about improvement. Initially, the KBs were focused largely on building relationships, but over time the knowledge translation role has gained importance. By the network’s fourth year the KBs were clearly seen as working collaboratively to create, test, and disseminate useful knowledge. Recently the KBs have begun to act as mentors who support groups that are launching new initiatives or improving their capacity to mobilize knowledge.

By the end of the fourth year, a reasonably clear picture of KB roles and skills had emerged (see Table 1). To support the development of the CoPs, to assist with specific initiatives, and to promote the growth of the network, SHRTN KBs have taken on a complex and demanding role. Moreover, the role is contextual. Supporting the development of a new CoP differs from supporting the efforts of a well-established CoP; assisting with an educational webinar differs from facilitating conversations among occupational groups that are suspicious of each other. These findings confirm the findings of others who suggest that the KB role is complex, diverse, and contextual [15,22,35,41].

**Table 1:** Roles and Skills of SHRTN KBs

**KB Roles**	**Related KB Skills and Attributes**
• **Coach and support** people and groups who are mobilizing activity toward goals, and facilitate processes of learning that allow clients to continually expand their capacity and improve their results.	• communication, adult education, experiential learning, facilitation, technology, flexibility, self-confidence
• Engage in **analytical and planning** activities to scan environments, assess resources and the readiness of groups to adopt, analyze data, and create strategies to help people achieve goals.	• working with groups, collaboration, organizational change, research, project management
• **Weave and expand networks** of people interested in similar issues and who might help each other to create and disseminate new approaches and solutions.	• communication, ability to influence and persuade, interpersonal skills, working with groups, collaboration, negotiation, ability to deal with ambiguity, persistence, self-confidence
• **Act as knowledge translators** by working with others to create, test, and disseminate documents and solutions.	• verbal and written communication, technical skills, facilitation, project management, research
• Help to **plan and facilitate** events at which experts and learners come together to discuss problems and solutions.	• communication, collaboration, empowerment, negotiation, facilitation, organizational change, adult education, ability to deal with ambiguity, flexibility, persistence, project management, organizational change
• When necessary help to **coordinate and manage** the administrative and logistical work of the communities they serve.	• communication, collaboration, negotiation, technical skills, project management, time management

In fact, a successful SHRTN KB may be asked to play roles associated with each of the three KB models posited by Ward and colleagues [22]. A single SHRTN KB may be asked to support one CoP by acting primarily as a knowledge translator, a second CoP by forging new relationships across organizational and occupational boundaries, and a third CoP by helping stakeholders learn to appraise knowledge resources.

Our findings are consistent with the KB roles and skills identified in the UK study reported by Ward and colleagues [22], and in the Canadian study reported by Dobbins and colleagues [15,35]. Our findings confirm that KBs act as: coaches and mentors to develop new skills and capacity; knowledge translators who locate, appraise, create, package, and disseminate knowledge to promote improvement and fill gaps; and developers of relationships and networks to bring people together into teams, communities and networks that focus on common problems.

The SHRTN evaluation findings, like the findings from the Canadian study [15,35], see an analytical and planning role for KBs. This role involves scanning the environment for resources, conducting assessments to identify needs and readiness for change, and developing strategies and plans to bring about change. The role also requires competence in organizational change that goes beyond project management skills, and includes sensitivity to organizational and occupational culture as barriers to or enablers for change. Like the Canadian study [15,35], our findings indicate that KBs often play an administrative role. In the case of SHRTN KBs, this involves handling coordination and logistical tasks, managing knowledge resources, and providing technical support.

The findings also show that SHRTN KBs are unique in that they are often called upon to work with partners to plan and facilitate events at which stakeholders assemble to discuss problems and brainstorm solutions. This role may have evolved due to the network’s overall purpose, which is to improve the health and health care of seniors in Ontario by bringing people together to discuss problems and share knowledge.

In SHRTN CoPs, a KB’s main objective is to facilitate access to the best available knowledge and evidence by connecting people to people, ideas and resources. Knowledge brokering is the human agency that enables the movement of knowledge from one place or group of people to another. Ultimately, knowledge brokering in SHRTN allows people to build relationships, uncover needs and share knowledge and ideas so the frontlines of healthcare have access to the best available evidence. SHRTN KBs facilitate the critical appraisal and adaptation of evidence-based material to particular user groups, monitor emerging trends and issues in the field, and respond to their CoP members’ ever-changing information and learning needs.

## Conclusions

Knowledge transfer is contextual. Knowledge brokering can be seen as a strategy that has developed in response to the unpredictable nature of knowledge flows within and across social systems. The SHRTN KB adapts to the social and technical affordances of each situation, and fashions a unique process to create relationships and promote learning and change. The SHRTN KB is an architect of information and conversations, an advocate of what is usable and useful, and a pragmatist. The SHRTN KB brings knowledge translation to life by assessing the urgency of the need for new solutions, the adaptability of different organizational contexts, and the facilitative processes available to bring about an exchange of knowledge.

This means that the ability to work with teams and to develop relevant models and approaches are critical KB skills. As facilitators, SHRTN KBs enable others to do for themselves, and thus help to create both new capacity in individuals and groups as well as new processes to foster improvement and change. The SHRTN KB resembles the new type of leader that is emerging in the literatures on leadership and social renewal, a leader who wields influence rather than power, and who is prepared to adopt whatever roles and approaches are needed to bring about a valuable result [55]. Seen in this light, it is perhaps not surprising that SHRTN KBs are unwilling to claim personal responsibility for achievements evident in the network. Their impact is on capacity and process, and the final outcomes that they pursue are best attributed to a team of women and men who together nurture a culture of collaboration.

While this analysis was based on our experience with SHRTN, we have seen growing interest in KBs in other Canadian knowledge networks and associations. At a March 2009 Think Tank on ‘Accelerating Knowledge Transfer and Exchange’ in Seniors’ Mental Health and Dementia, there was keen interest in KBs from participants from seven participating knowledge networks/organizations. The role of the KB is currently being explored by the Canadian Dementia Knowledge Translation Network (CDKTN) (http://www.lifeandminds.ca), one of the participating networks. The Canadian Dementia Resource and Knowledge Exchange team (http://www.dementiaknowledgebroker.ca), which is a component of the CDKTN, has highlighted a need for all individuals working within the health system to ‘think like knowledge brokers’ in order to achieve the successes that knowledge brokering can bring to all levels of the system [56,57]. Through an agreed-to set of KB core competencies, people throughout the continuum of care may function as KBs, improving evidence-informed decisions, reducing duplication and enhancing collaboration in ways that maximize shared resources and improve health outcomes. In this way, knowledge brokering may come to be seen less as a separate role than as a skill-set that would be helpful for policy-makers, caregivers, and researchers alike.

Whether we view knowledge brokering as a role to be taken on by specific individuals or as a function that must be spread throughout an organization or network, we must continue to investigate core KB competencies and skills. Like others, we call for more research on the way that knowledge brokering contributes to the success of knowledge translation, and for the development of educational programs based on a validated set of competencies and a body of relevant knowledge.

## Competing interests

Elizabeth Lusk and Megan Harris have both worked as knowledge brokers affiliated with SHRTN.

## Authors’ contributions

JC collaborated in the data gathering and analytical work, wrote the first draft of most sections of the article, and coordinated the revisions and review by the authoring team. MH and EL wrote first draft content for some sections, and read and commented on the draft. PS collaborated in the data gathering and analytical work, contributed content for the methods section, and read and commented on the draft. All authors read and approved the final manuscript.

## Supplementary Material

Additional file 1 Overall Evaluation Data Collection. Click here for file

Additional file 2 Data Pertaining to Knowledge Brokering. Click here for file

Additional file 3 The “doing” of knowledge brokering. Click here for file
