# Supplementary material for: Knowledge brokers in a knowledge network: the case of Seniors Health Research Transfer Network knowledge brokers
Source: Implement Sci. 2013 Jan 9;8:7. doi: 10.1186/1748-5908-8-7 (PMC3598713; doi:10.1186/1748-5908-8-7)
Supplement: Additional file 1 — Overall Evaluation Data Collection. [file 1748-5908-8-7-S1.doc]

## Additional file 1 – Overall Evaluation Data Collection

Year One

- A survey of advisory group members (n=9)
- Two surveys of network participants who attended a planning meeting in Toronto. The first (n=42) focused on participation in the network, and the second (n=31) focused on the activities that took place during the meeting.
- Observational data gathered at two face-to-face meetings
- Key informant interviews (n=13) carried out with leaders within Ontario’s seniors health sector
- A focus group (n=8) held with network champions
- Interim progress reports prepared by SHRTN partners (n=10)
- A survey of individuals (n=27) responsible for specific SHRTN deliverables
- A survey of stakeholders (n=27) who participated in a third network event
- Final reports submitted by SHRTN partner organizations

Year Two

- SHRTN contractual documents that outlined the agreement to establish and operate a library service for seniors health practitioners in Ontario.
- CoP agreements (n=11), Cop interim reports (n=11), and CoP final reports (n=11), and the SHRTN Board Orientation Guide.
- Reports submitted by the SHRTN central library service and regional library services (n=6).
- Interviews with the SHRTN librarians (n=6).
- Supplementary materials submitted by the SHRTN librarians to verify usage of the service and client satisfaction (e.g. session evaluation summaries, letters from users, patron feedback summaries)
- A survey of SHRTN KBs (n=4), with open-ended questions concerning the KB role, activities, successes and challenges, the importance of the KB role, and how that role could be made more effective (see Additional File 2).
- A survey of people who participated in a webinar on the issue of smoking cessation for people with dementia (n=6) that was sponsored by one of the SHRTN CoPs (this was part of the “test” of the proposed case study method, as were the following two activities)
- Interviews with the KB and CoP leads who participated in the webinar (n=4)
- Collection of background information and documentation on the issue of smoking cessation for people with dementia (10 documents concerning the issue and the plan for the webinar)

Year Three

- More than 20 SHRTN documents and reports, including the SHRTN strategic plan, Board of Directors Terms of Reference, Board Meeting Minutes, and other documents.
- Results from a detailed reporting template distributed to KBs (n=4) that asked KBs to provide details concerning their activities during the year, and lessons learned related to knowledge transfer and exchange.
- A case study focusing on a SHRTN CoP’s webinar series on driving and dementia. This case and the ones described below used standard case study methods [Creswell, 1998; Merriam, 1988; Patton, 2002; Stake, 1995]. Data were gathered through observations of the webinars, interviews with key informants (n=4), and a survey of session participants (n=40).
- A case study focusing on the work of a SHRTN CoP on continence care in long-term care facilities. Data were gathered through interviews (n=6), a focus group (n=12), and observations at six team meetings and learning sessions. In addition, the evaluators reviewed five interim and final reports produced by the CoP.
- A survey of Long-Term Care Administrators, Directors of Care, and Medical Directors in Ontario. Survey sample size was estimated using standard methods as described by Dillman [##]. The survey was sent to 473 potential respondents (drawn from a list provided by SHRTN); 137 responses were received, for a response rate of 29% (based on our previous work, we anticipated response rates for our evaluation surveys of ~25%).
- A survey of CoP leaders (again, the contact list was supplied by SHRTN). Once again, we used the Dillman formula to calculate sample size. The survey was sent to 201 members (randomly sampled); 59 responses were received, for a 29.4% response rate.

Years Four and Five

- A survey of representatives of 325 home and community care organizations serving Ontario seniors. Respondent lists were provided by the Ontario Association of Community Care Access Centres (n=66)), the Ontario Home Care Association (n=35), the Ontario Association of Non-Profit Homes and Services for Seniors (community members only, n=12), and the Ontario Community Support Association (n=212). The response rate was 20.4%.
- A survey of stakeholders at the SHRTN annual assembly (completed by 25 out of 101 participants). The survey gathered data about perceptions of SHRTN’s value, impact, and priorities.
- A focus group of KBs (n=5), interviews with CoP leaders who frequently work with KBs (n=12), and a review of documents related to the KB function.
- A survey of library users that elicited a 31% response rate (n=163). Data were also gathered through interviews with users of the library service (n=11), and through the library annual operating report.
- A focus group with SHRTN Secretariat staff (the people who provide administrative services for the overall network) (n=4).
- Activity and Aging CoP Case Study. Data included documents provided by the CoP, a group interview of CoP leaders (n=4), closing interviews with CoP leaders (n=2), field notes from seven webinars and meetings, and interviews with webinar participants (n=7).
- Aging and Developmental Disabilities CoP Case Study. Data included documents provided by the CoP, contexting interviews (n=4) with CoP leaders, interviews with people who played a leadership role in the case (n=7), and interviews with knowledge users (n=3).
- A review of more than 30 SHRTN documents, including the network’s annual report, the annual reports produced by 19 CoPs, and minutes from the monthly board meetings).
