# Supplementary material for: Knowledge brokers in a knowledge network: the case of Seniors Health Research Transfer Network knowledge brokers
Source: Implement Sci. 2013 Jan 9;8:7. doi: 10.1186/1748-5908-8-7 (PMC3598713; doi:10.1186/1748-5908-8-7)
Supplement: Additional file 2 — Data Pertaining to Knowledge Brokering. [file 1748-5908-8-7-S2.doc]

## Additional file 2 – Data Pertaining to Knowledge Brokering

Year 2 data pertaining to knowledge brokering:

- A survey of SHRTN KBs (n=4), with open-ended questions concerning the KB role, activities, successes and challenges, the importance of the KB role, and how that role could be made more effective (see Additional File 1).
- SHRTN contractual documents that outlined the agreement to establish and operate a library service for seniors health practitioners in Ontario.
- A survey of people who participated in a webinar on the issue of smoking cessation for people with dementia (n=6) that was sponsored by one of the SHRTN CoPs (this was part of the “test” of the proposed case study method, as was the following activity)
- Interviews with the KB and CoP leads who participated in the webinar (n=4)

Year 3 data pertaining to knowledge brokering:

- Results from a detailed reporting template distributed to KBs (n=4) that asked KBs to provide details concerning their activities during the year, and lessons learned related to knowledge transfer and exchange.
- SHRTN documents, including the SHRTN strategic plan, the network’s “2008-2010 Knowledge Exchange Strategy and Action Plan”, Board of Directors Terms of Reference, Board of Directors Meeting Minutes for ten meetings held in 2007-2008, and more than twenty other documents and reports.
- A case study focusing on a SHRTN CoP’s webinar series on driving and dementia (the case included data on the support provided by the KB assigned to the CoP). This case and the ones described below used standard case study methods [46-48]. Data was gathered through observations of the webinars, interviews with key informants (n=4), and a survey of session participants (n=40).
- A case study focusing on the work of a SHRTN CoP on continence care in long-term care facilities (the case included data on the support provided by the KB assigned to the CoP). Data was gathered through interviews (n=6), a focus group (n=12), and observations at six team meetings and learning sessions. In addition, the evaluators reviewed five interim and final reports produced by the CoP.
- A survey of CoP leaders (the contact list was supplied by SHRTN). Survey sample size was estimated using standard methods as described by Dillman [49]. The survey was sent to 201 members (randomly sampled); 59 responses were received, for a 29.4% response rate.

Year 4-5 data pertaining to knowledge brokering:

- A focus group of KBs (n=5), interviews with CoP leaders who frequently work with KBs (n=12), and a review of documents related to the KB function.
- A survey of stakeholders at the SHRTN annual assembly (completed by 25 out of 101 participants). The survey included 25 closed questions with a five-point Likert scale and 6 open questions, and gathered data about perceptions of SHRTN’s value, impact, and priorities.
- A focus group with SHRTN Secretariat staff (the people who provide administrative services for the overall network) (n=4).
- Activity and Aging CoP Case Study (the case included data on the support provided by the KB assigned to the CoP). Data included documents provided by the CoP, a group interview of CoP leaders (n=4), closing interviews with CoP leaders (n=2), field notes from seven webinars and meetings, and interviews with webinar participants (n=7).
- Aging and Developmental Disabilities CoP Case Study (the case included data on the support provided by the KB assigned to the CoP). Data included documents provided by the CoP, contexting interviews (n=4) with CoP leaders, interviews with people who played a leadership role in the case (n=7), and interviews with knowledge users (n=3).
- A review of more than 30 SHRTN documents, including the network’s annual report, the annual reports produced by 19 CoPs, and minutes from the monthly board meetings).
