# Supplementary material for: Knowledge brokers in a knowledge network: the case of Seniors Health Research Transfer Network knowledge brokers
Source: Implement Sci. 2013 Jan 9;8:7. doi: 10.1186/1748-5908-8-7 (PMC3598713; doi:10.1186/1748-5908-8-7)
Supplement: Additional file 3 — The “doing” of knowledge brokering. [file 1748-5908-8-7-S3.doc]

## Additional File 3 – The "doing" of knowledge brokering

During the network's fourth and fifth year of operation, the evaluation team was asked to provide a perspective on KBs do and what they have accomplished within SHRTN. We created four sets of narrative data to meet this requirement: interview responses from CoP leaders who were asked to comment on KB activities and accomplishments; interview responses from CoP leaders who were asked to identify the core skills needed by successful KBs; interview responses from CoP leaders who were asked about the ways that KBs facilitate the flow of knowledge through the network; and focus group data from KBs who were asked about their accomplishments. This additional file summarizes these findings.

When asked how SHRTN KBs support their CoPs, respondents focused on six areas.

1. KBs provide coordination and administrative support to CoPs by coordinating webinars and meetings, developing meeting materials, and managing the working documents of project teams. One person said, “There is a great deal of support from the KB in working with a core group to help prioritize activities and coordinate discussion, evaluate models of practice and current research, and to mediate various perspectives so that people are heard and their input is valued.”

2. KBs identify knowledge resources, and link CoP members with those resources. One CoP leader explained, “The KBs have been very helpful in providing an information flow, finding answers to questions that opinion leaders need information about, in a timely way, and they go back to ensure that the information has been useful.”

3. KBs create relationships and dialogue among people who may wish to work together. As one CoP leader said, “I have seen them bring people together around a common interest, ask the right questions, reach out to others when there are areas needing more understanding or a lack of coherence.”

4. KBs create linkages among CoPs that lead to partnerships and collaboration. One respondent said, “A SHRTN KB promotes and supports a CoP by connecting the members of the CoP to other CoP’s within the network…”

5. KBs promote the growth of new CoPs. One person said, “KBs are working to identify folks within a given area of practice to see if they are interested in coming together as a community.”

6. KBs possess skills and experience that lend them credibility and allow them to support the work of the CoPs. As one respondent explained, “…each of the KBs has had other professional skills that they have brought to their role. [KB name] was very skilled in health communication, and thus was able to help in a number of ways: providing advice around adapting existing educational materials to the CoP online modality and creating print materials (posters and evidence-based briefs).”

When asked to identify the skills of a successful KB, CoP leaders focused on four core skills.

1. A SHRTN KB must possess excellent verbal and written communication skills, including the ability to influence and persuade others.

2. They must be skilled at working with people and groups (including the ability to collaborate, direct, empower, support, and negotiate).

3. A KB must have subject matter and technical skills (facilitation, project management, organizational change, research, learning, and project management).

4. KBs must also possess personal qualities that equip them to work in ambiguous social environments (including time management, flexibility, and persistence).

CoP leaders were asked about the ways that KBs facilitate the flow of knowledge through the network. Respondents identified six areas of activity.

1. Analytical and planning activities such as searching for resources, assessing the usefulness of resources and the readiness of people to adopt innovations, analyzing data, and creating plans and strategies to help people achieve goals.

2. Coaching and supporting people who are mobilizing action toward goals, and facilitating processes of learning that allow stakeholders to expand their capacity and improve their results.

3. Weaving and expanding networks of people who are interested in similar issues and who help each other to create and disseminate new approaches and solutions.

4. Translating knowledge by creating, testing, and disseminating documents that offer solutions to relevant problems.

5. Planning and facilitating community events at which experts and learners come together to discuss problems and solutions.

6. Coordinating and managing the administrative and logistical work of the communities they serve.

When asked about their accomplishments, KBs minimized individual achievements and claimed that the KB team as a whole deserves credit for most accomplishments. Their responses yielded eight themes.

1. KBs exercise initiative to achieve awareness, understanding and definition of the role of KBs (including its emergent nature). One KB said, “The first thing that comes to mind is that we have developed a better understanding within this group about what it is to be a knowledge broker.” Another acknowledged that “Even with the strides we’ve made, it still is difficult to articulate what it is that we do.”

2. KBs draw on the value of experiential knowledge, and share that knowledge with each other and with others in SHRTN. One KB said, “Our group has something significant to offer. We have two nurses, one a psychiatric nurse and the other a ‘nurse’ nurse, and that’s great for me and, I suspect, for our other non-nursing knowledge brokers. ...I am richer for the experience of the different expertise of our team members.”

3. KBs create and maintain a repertoire of tools and approaches relevant for their practice. One KB said “We have built a repertoire of tools. ... CoP leads keep asking for templates. Knowledge brokers have to build the templates.”

4. KBs help loosely-coupled groups to generate concrete action around shared interests and goals. One KB explained, “...we know how to get a new group going, and how to help the CoP lead to decide what they want to accomplish.”

5. KBs provide information to SHRTN leaders to help them set a direction for the network. One KB explained that KBs help “to guide people like the SHRTN Executive Director in identifying what the network is lacking, or needs that must be met.”

6. KBs motivate, coordinate and organize. One KB spoke of participating on seven SHRTN committees, and acting as chair in most cases. Another KB said “You do active listening and coaching, and sometimes they just want to have somebody to talk to.”

7. KBs work as a team, and contribute to each other’s success. One KB told us that “...I don’t feel I can take 100% credit for any achievements. The ideas bounce off other people, and all of the processes and templates and documents that we have created have been a group effort.”

8. Despite all of this activity and success, some SHRTN members are not aware of the KBs. One KB acknowledged that “We still have some education to do with the network about our role in putting something like this together.”
